# Supplementary material for: Utilizing the SEIPS model to guide hand hygiene interventions at a tertiary hospital in Ethiopia
Source: PLoS One. 2021 Oct 28;16(10):e0258662. doi: 10.1371/journal.pone.0258662 (PMC8553035; doi:10.1371/journal.pone.0258662)
Supplement: S1 Appendix — (DOCX) [file pone.0258662.s001.docx]

| 3SOCIO-DEMOGRAPHIC AND JOB-RELATED CHARACTERISTICS | | | |
| --- | --- | --- | --- |
| **S No** | **Questions** | **Response** | **Skip** |
| **1** | Name of the unit you work on | 1.Medical 2. Surgical 3. Pediatrics  4. Gyn-Obs 5. OPD 6. Emergency 7. ICU |  |
| **2** | Age: | _____ in years |  |
| **3** | Sex: | 1. Male 2. Female |  |
| **4** | Marital status | 1.Single 2. Married 3. Divorce  4.Widow |  |
| **5** | Professional category | 1.Nurse 2 Midwives 3 Medical doctor 4. Resident 5. Medical student 6. Nursing student |  |
| **6** | Number of hours usually worked per week | 1.40 hours per week  2.> hours or more per week |  |
| **7** | Work hours (check the one that is most descriptive of the hours you work) | 1.Day 8-hour shift  2.Nights (12-hour shift)  3.Rotates between days and nights |  |
| **8** | Experience in your position: | _________in years |  |
| **9** | Did you receive training in hand hygiene in the last 3 years | 1. Yes  2.No |  |
| **10** | Was hand hygiene included in training when you were hired? | 1.Yes  2.No |  |
| **11** | Do you routinely perform hand hygiene | 1.Yes  2.No | If no go to 13A |
| **12** | If yes, what method do you use? | 1. Alcohol-based hand rub  2. Soap and water  3. Both |  |
| **13** | How often do you feel the unit staffing is adequate? | 1. Always 2. Sometimes 3. Rarely 4. Never |  |
| **14** | Salary | _______ in ETB |  |
| **15** | For how many patients do you care for during a normal shift? | ____ In number |  |

**S1 Appendix. Hand hygiene questionnaire.**

PART II A . Knowledge related questions

1. Which of the following is the main route of cross-transmission of potentially harmful germs between patients in a health-care facility? (*tick one answer only*)
2. Health-care workers’ hands when not clean
3. Air circulating in the hospital
4. Patients’ exposure to colonised surfaces (i.e., beds, chairs, tables, floors)
5. Sharing non-invasive objects (i.e., stethoscopes, pressure cuffs, etc.) between patients
6. What is the most frequent source of germs responsible for health care-associated infections?
   *(tick one answer only)*
7. The hospital’s water system
8. The hospital air
9. Germs already present on or within the patient
10. The hospital environment (surfaces)
11. Which of the following hand hygiene actions prevents transmission of germs *to the patient*?
12. Before touching a patient  Yes  No
13. Immediately after a risk of body fluid exposure  Yes  No
14. After exposure to the immediate surroundings of a patient  Yes  No
15. Immediately before a clean/aseptic procedure  Yes  No
16. Which of the following hand hygiene actions prevents transmission of germs *to the health-care worker*?
17. After touching a patient  Yes  No
18. Immediately after a risk of body fluid exposure  Yes  No
19. Immediately before a clean/aseptic procedure  Yes  No
20. After exposure to the immediate surroundings of a patient  Yes  No
21. Which of the following statements on alcohol-based hand rub and handwashing with soap and
    water are true?
22. Hand rubbing is more rapid for hand cleansing than handwashing 1. True 2. False
23. Hand rubbing causes skin dryness more than handwashing 1. True 2. False
24. Hand rubbing is more effective against germs than handwashing 1. True 2. False
25. Handwashing and hand rubbing are recommended to be performed in sequence 1. True 2. False
26. What is the minimal time needed for alcohol-based hand rub to kill most germs on your hands?
    *(tick one answer only)*
27. 20 seconds
28. 3 seconds
29. 1 minute
30. 10 seconds
31. Which type of hand hygiene method is required in the following situations?
32. Before palpation of the abdomen  Rubbing  Washing  None
33. Before giving an injection  Rubbing  Washing  None
34. After emptying a bedpan  Rubbing  Washing  None
35. After removing examination gloves  Rubbing  Washing  None
36. After making a patient's bed  Rubbing  Washing  None
37. After visible exposure to blood  Rubbing  Washing  None
38. Which of the following should be avoided, as associated with increased likelihood of colonisation of hands with harmful germs?
39. Wearing jewellery  Yes  No
40. Damaged skin  Yes  NO
41. Artificial fingernails  Yes  No
42. Regular use of a hand cream  Yes  No

**Part II B**

Attitude questions for hand hygiene

1. Strongly disagree 2. Disagree 3. Neutral 4. Agree 5. Strongly agree

| S.no | Questions | 1 | 2 | 3 | 4 | 5 |
| --- | --- | --- | --- | --- | --- | --- |
| 1 | I am tasked to act as a model about hand hygiene for other healthcare personnel. |  |  |  |  |  |
| 2 | It is more important for me to fulfill perfectly my tasks than doing hand hygiene when the given ward is busy |  |  |  |  |  |
| 3 | Execution of hand hygiene may reduce mortality of patients under the recommended conditions |  |  |  |  |  |
| 4 | Execution of hand hygiene may reduce the related medical costs to Nosocomial infections under the recommended conditions |  |  |  |  |  |
| 5 | I could not always do hand hygiene under the recommended situations because of preference of my patients’ requirements. |  |  |  |  |  |
| 6 | Prevention from the acquired infections is deemed as one of valuable roles for personnel of healthcare services. |  |  |  |  |  |
| 7 | I think one could follow the medical service officials in order to make decision for execution and or non- execution of hand hygiene |  |  |  |  |  |
| 8 | The existing infectious diseases in health care-giving environment may threaten my life and occupation. |  |  |  |  |  |
| 9 | I think I have potential to change poor performances regarding hand hygiene in my workplace. |  |  |  |  |  |
| 10 | The hand hygiene is assumed as a habit in my personal life. |  |  |  |  |  |
