# Supplementary material for: Utilizing the SEIPS model to guide hand hygiene interventions at a tertiary hospital in Ethiopia
Source: PLoS One. 2021 Oct 28;16(10):e0258662. doi: 10.1371/journal.pone.0258662 (PMC8553035; doi:10.1371/journal.pone.0258662)
Supplement: S2 Appendix — (DOCX) [file pone.0258662.s002.docx]

**S2 Appendix. Interview guide developed within the SEIPS model.**

Barriers and Facilitators to Hand Hygiene at JUMC Interview Guide

Date of interview:

Study Code:

What is hand hygiene?

Why do you think hand hygiene is important?

When do you do hand hygiene in the hospital?

Have you heard of the 5 moments for hand hygiene? Can you describe the 5 moments?

What are some things that make hand hygiene (cleaning your hands) difficult at JUMC?

What are some things that would make hand hygiene easier at JUMC?

How many patients do you care for in a usual day?

Does the patient’s symptoms or disease influence how you do hand hygiene?

Have you had formal training on proper hand hygiene? Can you describe the training? How often

have you had training?

Who provides education about hand hygiene at JUMC?

Did anyone teach you how to properly wash your hands with soap and water? Did anyone teach you how to use the alcohol hand rub?

Are there signs or posters explaining how to do hand hygiene at JUMC?

Is there anything that makes learning about hand hygiene difficult?

What would make learning about hand hygiene easier?

Is there an Infection Prevention and Control team at JUMC? Do you know any members?

Do you prefer using soap and water or alcohol hand rub to clean your hands? Why?

Is there anything that makes using soap and water difficult?

What would make using soap and water easier?

Is there anything that makes using alcohol hand rub difficult?

What would make using alcohol hand rub easier?

Are there enough sinks available to wash your hands?

Are sinks automated, handles, or foot pedals?

Is there enough water to wash your hands? Is there enough soap? Are there enough drying towels?

Are there enough alcohol hand rub dispensers in the hospital?

Are alcohol hand rub dispensers usually full?

Who is responsible for filling the dispensers?

Do you ever carry your own hand sanitizer?

Do you use gloves when caring for a patient? When do you use gloves?

When you use gloves, do you clean your hands before use? After use?

What makes glove use difficult?

What would make glove use easier?

Does someone monitor hand hygiene at JUMC?

Are hospital or ward rates posted?

Do you receive feedback about how you do hand hygiene?

Do you think your colleagues perform hand hygiene correctly?

Would you feel comfortable telling a colleague to do hand hygiene?

How many patients are in a room? How do you clean your hands between patients?
